# Ascites as the Presenting Symptom of Multiple Myeloma in a Scleroderma Patient

**DOI:** 10.1155/2014/235958

**Published:** 2014-11-20

**Authors:** Nadeen Hilal, Adnan Atallah

**Affiliations:** Department of Internal Medicine, Ain Wazein Hospital, P.O. Box 1503-2010/02, Ain Wazein, Lebanon

## Abstract

Several reports have demonstrated associations between scleroderma and cancer and between multiple myeloma and autoimmune diseases. Few papers have also reported the concurrence of scleroderma and multiple myeloma. We report a case of multiple myeloma that developed in a male patient after 28 years of fulfilling a diagnosis of scleroderma. The main presenting feature of multiple myeloma was ascites, solely explained by the increased vascular permeability that occurs in this disease.

## 1. Introduction

Scleroderma is a complex disease in which extensive fibrosis, vascular alterations, and autoantibodies against various cellular antigens are among the principal features [1]. The term refers to a heterogeneous group of autoimmune fibrosing disorders with morphea (localized scleroderma), limited cutaneous systemic sclerosis, diffuse cutaneous systemic sclerosis, and systemic sclerosis sine scleroderma encompassing the currently accepted disease subtypes [2].

The risk for cancer in patients with scleroderma is inconsistent among reports. Several studies have suggested that the risk is enhanced, with the most common cancers mentioned in these reports being lung cancers [3, 4]. Other types of reported cancers include breast cancer [3, 5], nonmelanoma skin cancer [3], non-Hodgkin's lymphoma [6], esophageal cancer [7, 8], and liver cancer [3]. A recent meta-analysis found that systemic sclerosis is associated with an increased risk of cancer, particularly lung, liver, hematologic, and bladder cancers, although absolute risk is relatively low [9]. In this meta-analysis, men with systemic sclerosis had a higher risk of developing cancer than women [9]. However, other studies have not demonstrated an increase in the risk of cancer among scleroderma patients [5, 10, 11].

Multiple myeloma (MM) is a neoplastic plasma-cell disorder that is characterized by clonal proliferation of malignant plasma cells in the bone marrow microenvironment, monoclonal protein in the blood or urine, and associated organ dysfunction [12, 13]. It accounts for approximately 1% of neoplastic diseases and 13% of hematologic cancers.

Multiple studies have evaluated the association between multiple myeloma and autoimmune diseases. In a retrospective cohort of more than 4 million white and black male United States veterans, the role of specific prior autoimmune, infectious, inflammatory, and allergic disorders in the etiology of MM and monoclonal gammopathy of undetermined significance (MGUS) was assessed. This study concluded that significantly elevated risks of MM were associated with broad categories of autoimmune disorders, thus concluding that various types of immune-mediated conditions might act as triggers for MM/MGUS development [14]. Another study examined the risk of myeloma systematically in patients who had been hospitalized for prespecified autoimmune diseases. This study concluded that standardized incidence ratio (SIR) for myeloma was significantly increased after ankylosing spondylitis (SIR 2.02) and systemic sclerosis (SIR 2.63) [15].

In this paper, we report a case of multiple myeloma that developed after 28 years of scleroderma diagnosis in a male, with ascites being the main presenting feature.

## 2. Case

A 66-year-old man was diagnosed 28 years ago to have diffuse scleroderma which manifested as diffuse skin tightness, telangiectasias, dysphagia, arthralgias, and Raynaud's phenomena. Disease had been quiet over many years with no worsening in any of his symptoms and no evidence of cardiac, pulmonary, or renal involvement. Thus, the patient was not receiving any specific treatment for scleroderma.

Few months prior to presentation, patient noticed change in his condition with fatigue, weight loss (20 kilograms in 4 months), and generalized feeling of not being well. At that time, patient did not seek any medical attention. Two weeks before presentation, patient developed progressive abdominal distention resulting in significant discomfort. Medical evaluation at presentation revealed normal vital signs, palor, and fluid in the abdomen, in addition to the findings of chronic scleroderma (diffuse tight skin, fish mouth appearance, and telangiectasias). There were no palpable masses or enlarged lymph nodes. Lungs were clear, heart exam was within normal limits with no murmurs or rubs, and there was no evidence of peripheral edema.

Patient underwent abdominal fluid tap. A sample of the ascitic fluid was sent for analysis. It revealed inflammatory cells with no suspicious malignant cells. Two weeks later, ascites recurred. At that time, patient was admitted to the hospital for full workup. Complete blood count revealed anemia with hemoglobin 10.9 g/dL and borderline white blood cell count (4.4 × 10^3^/*µ*L) and platelet count (176000/*µ*L). Lab tests also revealed hypercalcemia (10.3 mg/dL) with low albumin (31 g/L) and elevated globulin (50 g/L) levels. Thyroid function tests were normal. Renal parameters were also within normal limits. Patient underwent Echocardiography to assess cardiac function and rule out heart disease as the cause of ascites. Echocardiography revealed mild concentric hypertrophy, mild mitral valve and tricuspid valve regurgitation, and a mean pulmonary artery pressure of 35 mm Hg. Computed tomography of the chest, abdomen, and pelvis, performed to screen for malignancy, revealed marked abdominal and pelvic ascites with no masses or enlarged lymph nodes. Ascitic tap was repeated and fluid was sent again for analysis. Results revealed inflammatory cells with no malignant cells and negative culture results. Patient was started on spironolactone 50 milligrams daily and furosemide 40 milligrams daily.

**Figure 1:**
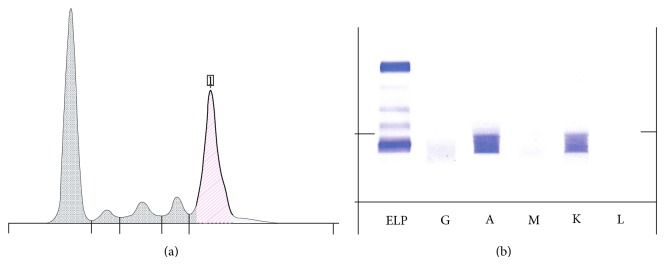
Protein electrophoresis. (a) Monoclonal peak in the Gamma zone. (b) IgA Kappa pattern.

Multiple myeloma was suspected based on anemia, hypercalcemia, and hyperglobulinemia, in addition to symptoms of weight loss and fatigue. Protein electrophoresis was done and showed monoclonal gammopathy. Immunofixation revealed IgA kappa pattern (Figure 1). Results of bone marrow biopsy revealed 45% plasma cells. Immunohistochemistry studies revealed a pattern consistent with multiple myeloma (strong expression of CD 138 in tumor cells).

A diagnosis of multiple myeloma was thus established. Further studies were obtained for disease staging. Beta-2-microglobulin level was 7.9 mg/L. X-ray skeletal series revealed osteopenia but no lytic lesions. Magnetic resonance imaging (MRI) of the abdomen, performed to assess the liver and spleen, failed to demonstrate any evidence of extramedullary hematopoiesis and thus failed to provide a clear explanation for ascites in this patient.

Patient was started on chemotherapy (Bortezomib, dexamethasone, and zoledronic acid). After six cycles of treatment, there was a major improvement in his disease condition with amelioration of anemia and normalization of globulin levels. Patient continued to have abdominal and pelvic fluid collection. However, the time interval separating the required abdominocentesis procedures increased gradually from around two weeks to around four weeks, suggesting a decrease in the quantity and rate of fluid collection. No change was noted in his scleroderma symptoms, neither worsening nor improvement.

## 3. Discussion

This case depicts the occurrence of two disease entities, scleroderma and multiple myeloma, despite a lag of 28 years. Several studies have shown an increased risk of cancer in general in scleroderma patients and an increased incidence of autoimmune diseases in multiple myeloma patients, as previously mentioned. The specific association between scleroderma and multiple myeloma has also been explored. The review of literature demonstrated that few articles have reported the cooccurrence of multiple myeloma and scleroderma [16] or scleroderma-like skin features [17–19]. Some cases have even experienced improvement of scleroderma symptoms or scleroderma-like skin changes after treatment with chemotherapy for multiple myeloma [16, 17].

As for the occurrence of ascites in this patient, it should be analyzed in the context of the two diseases, scleroderma and multiple myeloma. Scleroderma can cause ascites through few mechanisms, mainly liver involvement and portal hypertension, pulmonary hypertension, tricuspid regurgitation, or any pathology that results in decreased venous return to the heart such as constrictive pericarditis or right sided heart failure. All of these conditions have been ruled out in our patient by the appropriate screening methods. Multiple myeloma can also cause ascites. Effusions in general are relatively common in myeloma patients, affecting about 6% of patients, but are usually caused by sepsis, heart failure secondary to amyloidosis, hypoalbuminemia, or chronic renal failure rather than by myeloma cell involvement [20]. Myelomatous involvement of body cavity fluids is unusual, affecting less than 1% of patients [20], with pleural effusions occurring about twice as common as peritoneal effusions, while pericardial effusions are rare [21]. In our patient, MRI of abdomen and pelvis failed to reveal any evidence of extramedullary hematopoiesis. In addition, myeloma cells were not demonstrated in the aspirated fluid and all other etiologies for ascites were ruled out. Thus, a possible explanation for ascites in this patient is the increased vascular permeability that is thought to occur in multiple myeloma [22], resulting in fluid extravasation into the abdomen and pelvis.

## 4. Conclusion

This case and previously reported cases and studies suggest a possible association between scleroderma and multiple myeloma. The presentation of multiple myeloma is also atypical in this patient, with recurrent ascites being the main presenting symptom in the absence of any evidence of extramedullary hematopoiesis. With scleroderma being a relatively rare disease, large population based studies and registries are needed to determine whether there is a true increased risk of multiple myeloma in scleroderma patients. These studies may also help identify whether atypical features are present in patients who develop multiple myeloma with a background of scleroderma.

## References

[1] Medsger T. A., Koopman W. J. (1997). Systemic sclerosis (scleroderma): clinical aspects. *Arthritis and Allied Conditions: A Textbook of Rheumatology*.

[2] Fett N. (2013). Scleroderma: nomenclature, etiology, pathogenesis, prognosis, and treatments: facts and controversies. *Clinics in Dermatology*.

[3] Rosenthal A. K., McLaughlin J. K., Gridley G., Nyren O. (1995). Incidence of cancer among patients with systemic sclerosis. *Cancer*.

[4] Abu-Shakra M., Guillemin F., Lee P. (1993). Cancer in systemic sclerosis. *Arthritis and Rheumatism*.

[5] Duncan S. C., Winkelmann R. K. (1979). Cancer and scleroderma. *Archives of Dermatology*.

[6] Olesen A. B., Sværke C., Farkas D. K., Sørensen H. T. (2010). Systemic sclerosis and the risk of cancer: a nationwide population-based cohort study. *British Journal of Dermatology*.

[7] Derk C. T., Rasheed M., Artlett C. M., Jimenez S. A. (2006). A cohort study of cancer incidence in systemic sclerosis. *Journal of Rheumatology*.

[8] Kang K. Y., Yim H. W., Kim I. J., Yoon J. U., Ju J. H., Kim H. Y., Park S. H. (2009). Incidence of cancer among patients with systemic sclerosis in Korea results from a single centre. *Scandinavian Journal of Rheumatology*.

[9] Onishi A., Sugiyama D., Kumagai S., Morinobu A. (2013). Cancer incidence in systemic sclerosis: meta-analysis of population-based cohort studies. *Arthritis and Rheumatism*.

[10] Chatterjee S., Dombi G. W., Severson R. K., Mayes M. D. (2005). Risk of malignancy in scleroderma: a population-based cohort study. *Arthritis and Rheumatism*.

[11] Thomas E., Brewster D. H., Black R. J., Macfarlane G. J. (2000). Risk of malignancy among patients with rheumatic conditions. *International Journal of Cancer*.

[12] Kyle R. A., Rajkumar S. V. (2004). Drug therapy: multiple myeloma. *The New England Journal of Medicine*.

[13] Kyle R. A., Rajkumar S. V. (2005). *The New England Journal of Medicine*.

[14] Brown L. M., Gridley G., Check D., Landgren O. (2008). Risk of multiple myeloma and monoclonal gammopathy of undetermined significance among white and black male United States veterans with prior autoimmune, infectious, inflammatory, and allergic disorders. *Blood*.

[15] Hemminki K., Liu X., Forsti A., Ji J., Sundquist J., Sundquist K. (2012). Effect of autoimmune diseases on incidence and survival in subsequent multiple myeloma. *Journal of Hematology and Oncology*.

[16] Čolović M., Jurisic V., Bila J., Čolović N., Palibrk V. (2011). FGF-R3 and OPG expression in patient with multiple myeloma following systemic sclerosis: case report and review of the literature. *International Journal of Hematology*.

[17] Santos-Juanes J., Galache Osuna C., Curto Iglesias J. R., De Quiros J. F. B., Sánchez Del Río J. (2001). Treatment with chemotherapy of scleredema associated with Ig A myeloma. *International Journal of Dermatology*.

[18] Casper C., Scharffetter-Kochanek P., Bohlen H., Linke R. P., Krieg T., Hunzelmann N. (1999). Light chain multiple myeloma with peripheral leucocytosis presenting as scleroderma amyloidosum of the A*λ*-type. *British Journal of Dermatology*.

[19] Paredes-Suárez C., Fernández-Redondo V., Vázquez Blanco M., Sánchez-Aguilar D., Toribio J. (2005). Multiple myeloma with scleroderma-like changes. *Journal of the European Academy of Dermatology and Venereology*.

[20] Manley R., Monteath J., Patton W. N. (1999). Co-incidental presentation of IgA lambda multiple myeloma and pleural involvement with IgM kappa non-Hodgkin's lymphoma. *Clinical and Laboratory Haematology*.

[21] Sasser R. L., Yam L. T., Li C.-Y. (1990). Myeloma with involvement of the serous cavities. Cytologic and immunochemical diagnosis and literature review. *Acta Cytologica*.

[22] Young P., Finn B. B., Pellegrini D., Bruetman J. E., Shanley C. M., Tolosa Vilell C., Trimarchi H. (2008). Myelomatous ascites. *Anales de Medicina Interna*.

